# Toe pressure and toe brachial index are predictive of cardiovascular mortality regardless of the most diseased arterial segment in symptomatic lower-extremity artery disease—A retrospective cohort study

**DOI:** 10.1371/journal.pone.0259122

**Published:** 2021-11-15

**Authors:** V. Koivunen, M. Juonala, M. Venermo, M. Laivuori, J. M. Jalkanen, H. H. Hakovirta

**Affiliations:** 1 Faculty of Medicine, University of Turku, Turku, Finland; 2 Department of Internal Medicine, University of Turku, Turku, Finland; 3 Division of Medicine, Turku University Hospital, Turku, Finland; 4 Department of Vascular Surgery, Abdominal Center, Helsinki University Hospital and University of Helsinki, Helsinki, Finland; 5 Department of Vascular Surgery, Turku University Hospital, University of Turku, Turku, Finland; 6 Department of Surgery, Satakunta Central Hospital, Pori, Finland; Medical University Innsbruck, AUSTRIA

## Abstract

**Objective:**

Although lower extremity arterial disease (LEAD) is most often multisegmental, the predominant disease location and risk factors differ between patients. Ankle-brachial index (ABI), toe-brachial index (TBI), and toe pressure (TP) are predictive of outcome in LEAD patients. Previously, we reported a classification method defining the most diseased arterial segment (MDAS); crural (CR), femoropopliteal (FP), or aortoiliac (AOI). Current study aimed to analyze the associations between MDAS, peripheral pressure measurements and cardiovascular mortality.

**Materials and methods:**

We reviewed retrospectively 729 consecutive LEAD patients (Rutherford 2–6) who underwent digital subtraction angiography between January, 2009 to August, 2011 and had standardized peripheral pressure measurements.

**Results:**

In Cox Regression analyses, cardiovascular mortality was associated with MDAS and non-invasive pressure indices as follows; MDAS AOI, TP <30 mmHg (HR 3.00, 95% CI 1.13–7.99); MDAS FP, TP <30 mmHg (HR 2.31, 95% CI 1.36–3.94), TBI <0.25 (HR 3.20, 95% CI 1.34–7.63), ABI <0.25 (HR 5.45, 95% CI 1.56–19.0) and ≥1.30 (HR 6.71, 95% CI 1.89–23.8), and MDAS CR, TP <30 mmHg (HR 4.26, 95% CI 2.19–8.27), TBI <0.25 (HR 7.71, 95% CI 1.86–32.9), and ABI <0.25 (HR 2.59, 95% CI 1.15–5.85).

**Conclusions:**

Symptomatic LEAD appears to be multisegmental with severe infrapopliteal involvement. Because of this, TP and TBI are strongly predictive of cardiovascular mortality and they should be routinely measured despite the predominant disease location or clinical presentation.

## Introduction

Lower-extremity arterial disease (LEAD), both symptomatic and asymptomatic, is associated with elevated risk for cardiovascular and all-cause mortality [1]. Chronic limb-threatening ischemia (CLTI), the most severe form of LEAD, is related with even higher risk of death [1]. Early detection of LEAD is crucial since maximal medical intervention can significantly reduce LEAD related morbidity and mortality [2].

Current guidelines recommend ankle-brachial index (ABI), the highest systolic ankle pressure divided by the highest systolic brachial pressure, as the first line test for screening and diagnosis of LEAD [1]. Vascular calcification, commonly seen in patients with diabetes mellitus (DM) and chronic kidney disease (CKD), can falsely elevate ABI [3, 4]. In these cases, toe pressure (TP) and toe brachial index (TBI) are recommended [1]. Both ABI ≤0.90 and >1.40 have been associated with increased risk for cardiovascular and overall mortality [5, 6]. Poor TBI has been associated with increased mortality and major adverse cardiovascular events although the studies are mainly investigating patients with DM and renal insufficiency [7–9]. Similarly, in unselected LEAD cohort poor TP has been reported to be predictive of cardiovascular mortality [10, 11].

Extensive atherosclerosis in different arterial segments, aortoiliac (AOI), femoropopliteal (FP), and crural (CR), has been noted to associate with distinctive risk factors and disease etiology [12–15]. Majority of the LEAD patients have atherosclerotic lesions in several arterial segments instead of isolated atherosclerosis in one arterial segment [14, 16]. We have recently created a new classification system, Crural Index (CIx), in order to grade the lower limb atherosclerosis and determine the most diseased arterial segment (MDAS) [17, 18]. In previous studies, we have shown the association between this classification system, cardiovascular mortality and amputation free survival [17, 18].

Previously in one study, a decline in ABI has been interpreted as disease progression in large-vessels when a reduction in TBI has been associated with progression in small vessel disease [12]. As atherosclerosis in different anatomical segments associates with different risk factors and hemodynamic parameters, it could be assumed that the outcome predictors would also vary according to MDAS. Less evidence exists regarding the most appropriate peripheral pressure measurement for each MDAS to detect the patients with the greatest risk of death. Therefore, we aimed to investigate the association between non-invasive pressure parameters, MDAS, and cardiovascular mortality.

## Materials and methods

### Study cohort

This is a retrospective patient cohort study. Irrespective of earlier vascular history or procedures, all patients (887) at the Turku University Hospital vascular unit who underwent digital subtraction angiography (DSA) from January 1, 2009 to August 31, 2011, were recruited to the study. Of these (Rutherford 2–6), 729 patients (82.2%) who both underwent DSA and had standardized peripheral pressure measurement in vascular laboratory were included for the final analyses. Patients were subjected to DSA for either diagnostic or intention-to-treat reasons. Within the recruitment, DSA was the gold standard imaging modality to visualize atherosclerotic changes in lower limb arteries at our vascular department. The limb with the lowest average TP was selected to the study. If repetitive DSAs were performed over the study period, only the first DSA was included in the study.

### Data processing

The demographic patient data was retrospectively collected from the hospital electronic operations database. The date of DSA served as the index date for the survival. The comorbidities registered according to the International Statistical Classification of Diseases and Related Health Problems classification system (ICD-10) were collected as follows; coronary artery disease (CAD), hypertension (HT), end stage renal disease (ESRD), dyslipidemia, and atrial fibrillation (AF). diabetes (DM), cerebrovascular disease (CVD), sleep apnea, and chronic obstructive pulmonary disease (COPD).

The baseline medication (statins, antithrombotic or anticoagulation), severity of ischemia (Rutherford classification [19]) and smoking history were registered from electronic patient records. The date and cause of death were collected from the Causes of Death Registry of Statistics Finland [20]. ICD-10 codes of I00-I42.5 and I42.7-I99 were considered as cardiovascular causes of death.

### Peripheral pressure measurements

Hemodynamic measurements were conducted with Nicolet VasoGuard (Nicolet Vascular Inc. Madison, WI, USA) photoplethysmography in Turku University Hospital Vascular Laboratory. All assessments were standardized and obtained in a systematic manner; patients were in supine position with the limb at the level of the heart. To obtain limb pressures, pneumatics cuffs were inflated until the flow signal ceased and then slowly deflated until the pulsatile signal reappeared. Overall, the lowest value of the pressure measurement was registered.

To assess ABI, the systolic ankle pressure was divided by the higher systolic brachial pressure. TP was assessed from the great toe and in case it was missing, from the nearest available toe. To reduce the effect of local vasoconstriction, the patients’ feet were pre-warmed before the measurements. TBI was measured by dividing the toe’s systolic pressure by the brachial systolic pressure.

According to the TransAtlantic Inter-Society Consensus II (TASC II) classification on CLTI, peripheral pressure measurements were then categorized [21]. For TP, classification was <30 mmHg, 30–49 mmHg, and ≥50 mmHg and for TBI, <0.25, 0.25–0.49, and ≥0.50. For ABI, <0.25, 0.25–0.89, 0.90–1.29, and ≥1.30, respectively.

### Classification of MDAS

First, all DSA images were analyzed to assess MDAS. This process of the determination of MDAS was initially introduced by Jalkanen et al. [18]. All the images were primarily analyzed by a single observer and cross-checked by another observer. Both observers were vascular surgeons.

AOI and FP segments were classified according to the TASC II criteria. To assess the state of atherosclerosis in each segment, AOI and FP segments were coded as follows; no disease:0, TASC II A: 1; TASC II B: 2; TASC II C: 3 and TASC II D: 4. In CR segment, each of the three CR vessels were analyzed and coded individually as follows: No detectable or minor disease: 0; Total occlusion less than 5 cm: 1; Total occlusion less than 15 cm: 3; Total occlusion more than 15 cm: 4. Finally, the Crural Index (CIx) was then assessed by a sum of the these three CR vessels: if the sum was 0 the CIx was 0; if the sum was 1–3 the CIx was I (1); if the sum was 4–6 the CIx was II (2); if the sum was 7–9 the CIx was III (3) and if the sum was 10–12, the CIx was IV (4).

Based on TASC II and CIx classifications, each segment was scored from 0–4. The arterial segment reaching the highest score was defined as MDAS. According to this, patients were assigned into either 1) MDAS AOI, 2) MDAS FP, or 3) MDAS CR group. For instance, CIx III (3) ruled out proximal lesions of 0–2 and therefore, the predominant disease location was CR and patients were analyzed in the group MDAS CR. As an exception, the most proximal location was chosen if the highest grade was equal in two or even three locations.

### Ethical considerations

The study protocol was approved by the local ethical committee of the Hospital District of Southwest Finland with a decision ID TK-53-1266-15 and due to the retrospective nature of the present study, no informed consent was required. This study conforms to the ethical guidelines of the 1975 Declaration of Helsinki.

### Statistical analyses

Statistical analyses were performed using the IBM SPSS ® version 26 statistics program. Shapiro-Wilk test was used to test the normality. Continuous variables were expressed as mean ± standard deviation (SD) and Kruskal-Wallis test was used for comparisons. Categorical variables were expressed as frequency and percentage and comparisons were performed by Chi-square test. A Cox regression analysis was performed to assess the predictive value of factors affecting survival. The following confounding variables were added to the model; age, male sex, CAD, hypertension, DM, smoking, and ESRD. In Multivariate Cox regression analyses, cardiovascular cause of death was considered as the outcome. The highest group was selected as reference for categorical pressures and indices except ABI, where ABI 0.90–1.29 were selected as reference category. Survival curves were estimated for each group using the Kaplan-Meier method and compared statistically using the log-rank statistics. To estimate the validity of the findings, the calculation of sample size was based on the cardiovascular mortality at 3-years with the assumption of the alpha-level of 0.05. To achieve a power of 80% (beta-error 0.2) with TP <30 mmHg, sample size calculation resulted in 69 patients. Correspondingly, the calculated sample size was 84 for TBI <0.25 and 75 for ABI <0.25. All authors had full access to all the data in the study. The corresponding author takes responsibility for the integrity of the data analyses. Data cannot be shared publicly because of patient identification. Data are available from the corresponding author for researchers who meet the criteria for access to confidential data.

## Results

### Characteristics

Demographic characteristics overall and according to MDAS are presented in Table 1. Mean age of the patients was 74.9 (±10.5) years and 40.9% had diabetes. In Table 2., the lesion characteristics according to TASC II and CIx classifications for each MDAS are presented.

**Table 1:** Cohort characteristics according to MDAS.

	MDAS AOI N (%, SD)	MDAS FP N (%, SD)	MDAS CR N (%, SD)	All N (%, SD)	*p*
N of patients	129	367	233	729	
Age in years	72.8 (±10.4)	74.4 (±10.0)	76.8 (±11.2)	74.9 (±10.5)	<0.001
Men	87 (67.4)	215 (58.6)	124 (53.2)	426 (58.4)	0.031
Rutherford 0–3	88 (68.2)	192 (52.3)	52 (22.3)	332 (45.5)	<0.001
Rutherford 4–6	41 (31.8)	175 (47.7)	181 (77.7)	397 (54.5)	<0.001
DM	30 (23.3)	144 (39.2)	124 (53.2)	298 (40.9)	<0.001
CAD	55 (42.6)	161 (43.9)	99 (42.5)	315 (43.2)	0.939
CVD	20 (15.5)	64 (17.4)	38 (16.3)	122 (16.7)	0.879
HT	88 (68.2)	249 (67.8)	169 (72.5)	506 (69.4)	0.456
COPD	23 (17.8)	57 (15.5)	11 (4.7)	91 (12.5)	<0.001
Sleep apnea	6 (4.7)	23 (6.3)	15 (6.4)	44 (6.0)	0.817
ESRD	8 (6.2)	30 (8.2)	30 (12.9)	68 (9.3)	0.068
Dyslipidemia	52 (40.3)	152 (41.4)	69 (29.6)	273 (37.4)	0.010
AF	19 (14.7)	84 (22.9)	58 (24.9)	161 (22.1)	0.066
Antithrombotic medication	104 (80.6)	261 (71.1)	146 (62.7)	511 (70.1)	0.006
Statin	76 (58.9)	212 (57.8)	125 (53.6)	413 (56.7)	0.833
Anticoagulation	19 (14.7)	84 (22.9)	58 (24.9)	161 (22.1)	0.045
Smoking history	68 (52.7)	121 (33.0)	23 (9.9)	212 (29.1)	<0.001

For continuous variables, p-value is calculated with Kruskal-Wallis test and for categorial variables, with Fisher’s Exact test.

MDAS, most diseased arterial segment; AOI, aortoiliac; FP, femoropopliteal; CR, crural; N, number; SD, standard deviation; DM, diabetes mellitus; CAD, coronary artery disease; CVD, cerebrovascular disease; HT, hypertension; COPD, chronic obstructive pulmonary disease; AF, atrial fibrillation.

**Table 2:** Lesion characteristics according to MDAS.

		MDAS AOI N (%)	MDAS FP N (%)	MDAS CR N (%)	All (%)	*p*
N		129	367	233	729	
TASC II AOI	0	0	300 (81.7)	207 (88.8)	507 (69.5)	<0.001
	A-B	64 (49.6)	53 (14.4)	20 (8.6)	137 (18.8)	<0.001
	C-D	65 (50.4)	14 (3.8)	6 (2.6)	85 (11.7)	<0.001
TASC II FP	0	63 (48.8)	0	109 (46.8)	172 (23.6)	<0.001
	A-B	30 (23.3)	87 (23.7)	73 (31.3)	190 (26.1)	0.111
	C-D	36 (27.9)	280 (76.3)	51 (21.9)	367 (50.3)	<0.001
CIx	0	40 (31.0)	74 (20.2)	0	114 (15.6)	<0.001
	I-II	59 (45.7)	164 (44.7)	48 (20.6)	271 (37.2)	<0.001
	III-IV	30 (23.3)	129 (35.1)	185 (79.4)	344 (47.2)	<0.001

For categorial variables, p-value is calculated with Fisher’s Exact test.

MDAS, most diseased arterial segment; AOI, aortoiliac; FP, femoropopliteal; CR, crural; N, number; TASC II, TransAtlantic Inter-Society Consensus II; CIx, Crural Index.

### TP and pressure indices

Number of patients according to TP, TBI and ABI and MDAS are presented in Table 3. ABI measurements were available for 720/729 (98.8%) and TP for 714/729 (97.9%) and TBI for 712/729 patients (97.7%). Measurements were lacking due to loss of co-operation, painful CR wounds, missing toe or toes, or gangrene.

**Table 3:** Number of patients according to TP, TBI and ABI in the three groups based on MDAS.

		MDAS AOI N (%)	MDAS FP N (%)	MDAS CR N (%)	All N (%)	*p*
	N total	129	367	233	729	
TP (mmHg)	<30	33 (25.6)	107 (29.2)	87 (37.3)	227 (31.1)	0.021
	30–49	36 (27.9)	116 (31.6)	78 (33.5)	230 (31.6)	0.478
	≥50	57 (44.2)	141 (38.4)	59 (25.3)	257 (35.3)	0.001
TBI	<0.25	39 (30.2)	128 (34.9)	100 (42.9)	267 (36.6)	0.017
	0.25–0.49	66 (51.2)	184 (50.1)	102 (43.8)	352 (48.3)	0.397
	≥50	21 (16.3)	52 (14.2)	20 (8.6)	93 (12.8)	0.066
ABI	<0.25	9 (7.0)	33 (9.0)	18 (7.7)	60 (8.2)	0.788
	0.25–0.89	114 (88.4)	284 (77.4)	137 (58.8)	535 (73.4)	<0.001
	0.90–1.29	4 (3.1)	22 (6.0)	28 (12.0)	54 (7.4)	0.003
	≥1.30	1 (0.8)	23 (6.3)	47 (20.2)	71 (9.7)	<0.001

P-value is calculated with Fisher’s Exact test.

MDAS, most diseased arterial segment; AOI, aortoiliac; FP, femoropopliteal; CR, crural; N, number; AP, ankle pressure; TP, toe pressure; TBI, toe-brachial index; ABI, ankle-brachial index.

### TP and pressure indices as risk factors for mortality

Altogether, 28.8% (N = 210) patients died due to cardiovascular causes during the follow up; 12.4% in MDAS AOI (N = 26), 40.0% in MDAS FP (N = 84), and 47.6% in MDAS CR (N = 100) (*p*<0.001). For MDAS AOI, 1-, 2- and 3-years survival was 96.0%, 96.0%, and 89.1%, respectively. For MDAS FP, the corresponding proportions were 93.4%, 88.2%, and 84.9%, and for MDAS CR, 81.9%, 71.3%, and 63.1%, respectively.

In Table 4., risk factors for poor survival according to Multivariate Cox regression analyses are presented. The following confounding variables were added to the model; age, male sex, CAD, hypertension, DM, smoking, and ESRD. Due to the small number of cases in ABI categories of <0.25 (N = 9), 0.25–0.89 (N = 4), and ≥1.30 (N = 1), HR, 95% CI and *p*-value were only available for patients with ABI 0.9–1.29 in MDAS AOI.

**Table 4:** Multivariate Cox regression analyses for cardiovascular mortality according to MDAS.

	MDAS	MDAS AOI (HR, 95% CI)	*p*	MDAS FP (HR, 95% CI)	*p*	MDAS CR (HR, 95% CI)	*p*
TP (mmHg)	<30	3.00 (1.13–7.99)	0.027	2.31 (1.36–3.94)	0.002	4.26 (2.19–8.27)	<0.001
	30–49	0.83 (0.29–2.41)	0.735	1.34 (0.76–2.38)	0.315	2.55 (1.28–5.08)	0.008
	≥50	Reference		Reference		Reference	
TBI	<0.25	2.40 (0.71–8.09)	0.158	3.20 (1.34–7.63)	0.009	7.71 (1.86–32.1)	0.005
	0.25–0.49	1.14 (0.35–3.71)	0.833	1.96 (0.82–4.67)	0.128	4.67 (1.12–19.5)	0.035
	≥0.50	Reference		Reference		Reference	
ABI	<0.25	NA	NA	5.45 (1.56–19.0)	0.008	2.59 (1.15–5.85)	0.022
	0.25–0.89	NA	NA	1.86 (0.57–6.04)	0.305	1.16 (0.61–2.19)	0.648
	0.90–1.29	Reference		Reference		Reference	
	≥1.30	NA	NA	6.71 (1.89–23.8)	0.003	1.08 (0.50–2.32)	0.852

Note. The following confounding variables have been added to the model; age, male sex, CAD, hypertension, DM, smoking, and ESRD.

MDAS, most diseased arterial segment; AOI, aortoiliac; FP, femoropopliteal; CR, crural; HR, hazard ratio; CI, confidence interval; TP, toe pressure; TBI, toe-brachial index; ABI, ankle-brachial index.

### Survival—Toe pressure (TP)

**Fig 1:**
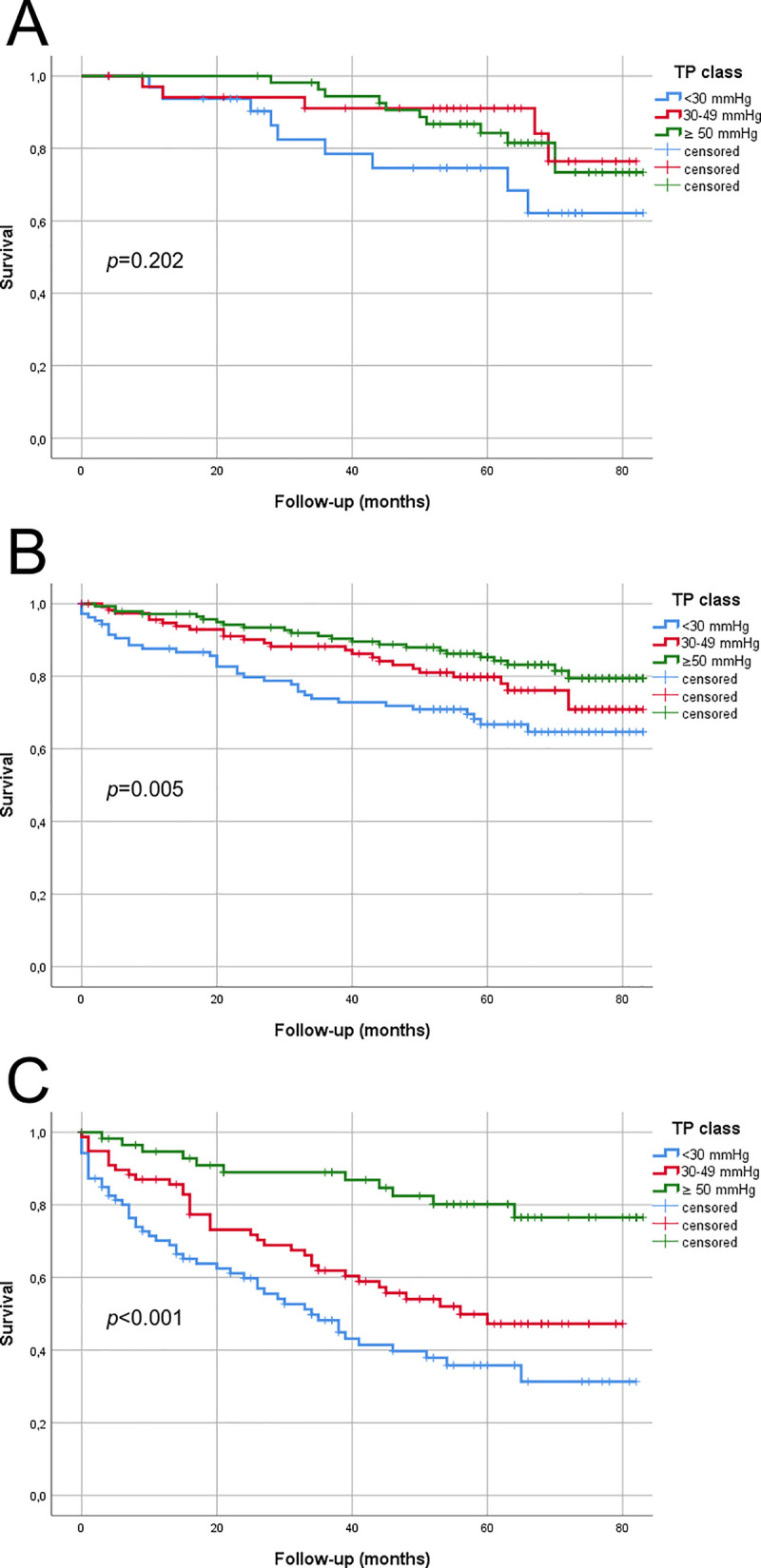
Kaplan Meier estimated survival within TP groups according to MDAS. A) MDAS AOI, B) MDAS FP, and C) MDAS CR. P-value is calculated with the log-rank statistics. A total of 129 patients were in MDAS AOI, 367 in MDAS FP, and 233 in MDAS CR group.

The Kaplan Meier estimated survival in the three TP groups is illustrated in the Fig 1. For MDAS AOI, the mean survival for TP<30 mmHg was 66.5 months (SE 4.7, 95% CI 57.3–75.5), for TP 30–49 mmHg 74.3 months (SE 3.3, 95% CI 67.7–80.8), and for ≥50 mmHg 75.3 months (SE 2.1, 95% CI 71.1–79.4). For MDAS FP, the mean survival for TP <30 mmHg was 62.6 months (SE 3.0, 95% CI 56.8–68.5), for TP 30–49 mmHg 70.6 months (SE 2.3, 95% CI 66.1–75.0), and for ≥50 mmHg 74.4 months (SE 1.7, 95% CI 70.9–77.8). For MDAS CR, the mean survival for <30 mmHg was 40.6 months (SE 3.8, 95% CI 33.2–47.9), for 30–49 mmHg 51.2 months (SE 3.6, 95% CI 44.3–58.3), and for ≥50 mmHg 71.2 months (SE 3.3, 95% CI 64.7–77.6).

### Survival—Toe-brachial index (TBI)

**Fig 2:**
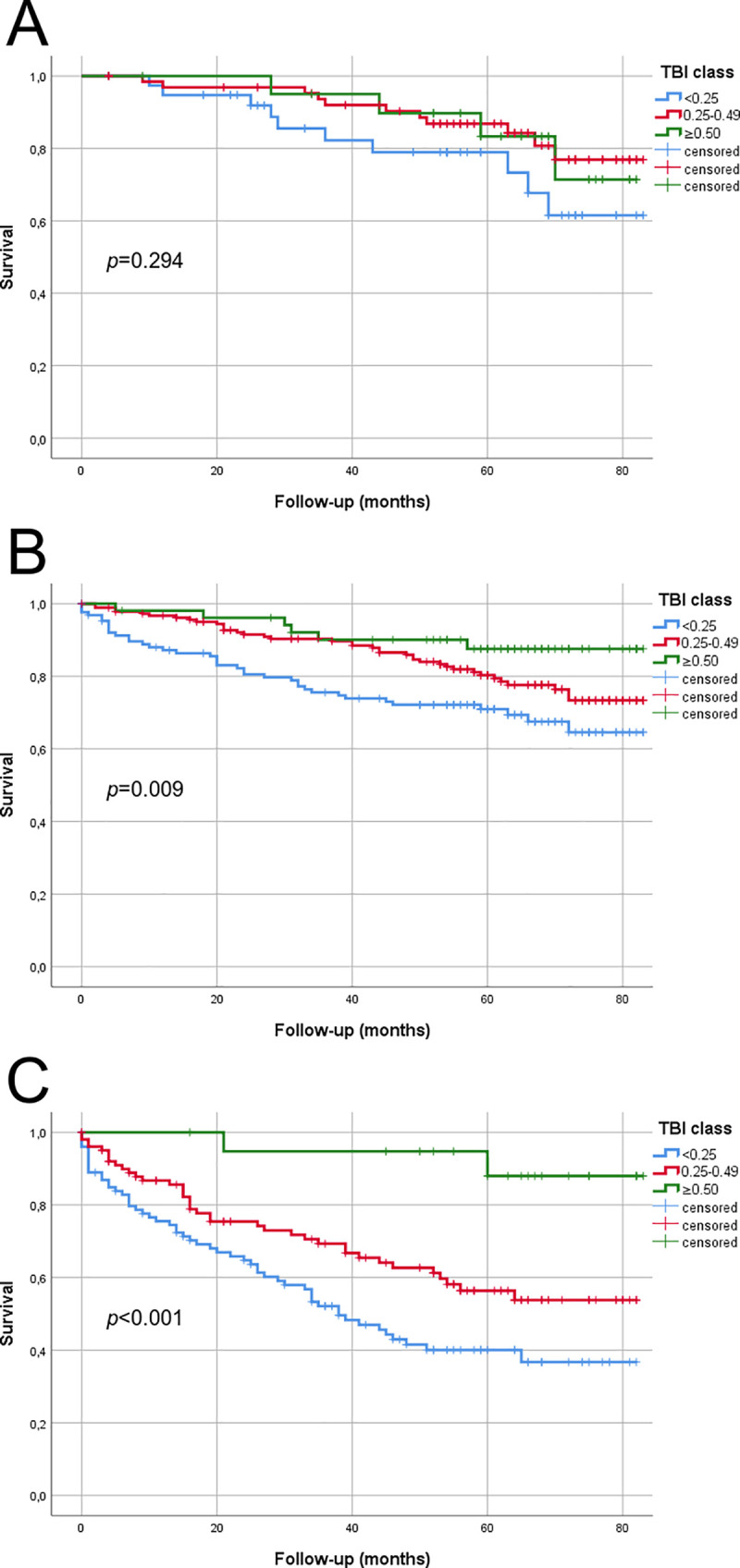
Kaplan Meier estimated survival within TBI groups according to MDAS. A) MDAS AOI, B) MDAS FP, and C) MDAS CR. P-value is calculated with the log-rank statistics. A total of 129 patients were in MDAS AOI, 367 in MDAS FP, and 233 in MDAS CR group.

The Kaplan Meier estimated survival within TBI groups is presented in Fig 2. For MDAS AOI, the mean survival for TBI <0.25 was 68.3 months (SE 4.0, 95% CI 60.4–76.2), for TBI 0.25–0.49 75.0 months (SE 2.3, 95% CI 70.5–79.5), and for TBI ≥0.50 74.4 months (SE 3.5, 95% CI 67.5–81.3). For MDAS FP, the mean survival for was TBI <0.25 63.6 months (SE 2.7, 95% CI 58.2–68.9), for TBI 0.25–0.49 72.0 months (SE 1.7, 95% CI 68.7–75.3), and for TBI ≥ 50 76.5 months (SE 2.6, 95% CI 71.4–81.5). For MDAS CR, the mean survival for TBI <0.25 was 44.2 months (SE 3.4, 95% CI 37.5–50.9), for TBI 0.25–0.49 56.2 months (SE 3.3, 95% CI 49.7–62.6), and for TBI ≥50 78.2 months (SE 3.4, 95% CI 71.4–84.9).

### Survival—Ankle-brachial index (ABI)

**Fig 3:**
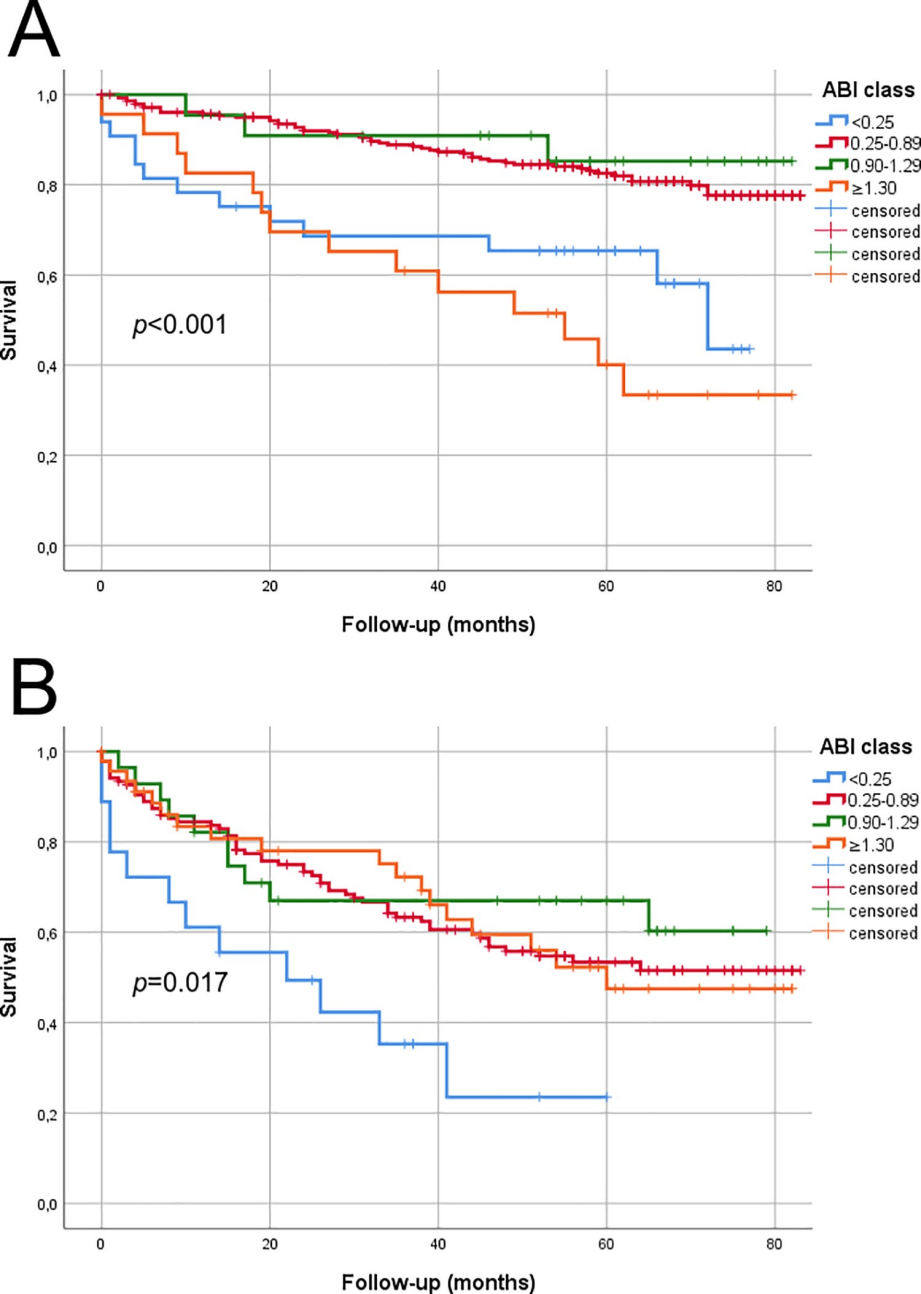
Kaplan Meier estimated survival within ABI groups according to MDAS. A) MDAS FP, and B) MDAS CR. Due to the small number of cases in ABI categories except for normal ABI 0.9–1.29, no reliable Kaplan Meier estimation was available for MDAS AOI. P-value is calculated with the log-rank statistics. A total of 367 were in MDAS FP and 233 in MDAS CR group.

In Fig 3, the Kaplan Meier estimated survival within ABI groups is illustrated. Due to the small number of cases in ABI categories of <0.25 (N = 9), 0.25–0.89 (N = 4), and ≥1.30 (N = 1), except for 0.90–1.29 (N = 114), no reliable Kaplan Meier estimation was available for MDAS AOI. For MDAS FP, the mean survival for ABI <0.25 was 52.9 months (SE 5.6, 95% CI 42.0–63.8), for ABI 0.25–0.89 72.7 months (SE 1.4, 95% CI 70.0–75.4), for ABI 0.9–1.29 74.1 months (SE 4.4, 95% CI 65.5–82.7), and for ABI ≥1.30 48.4 months (SE 6.2, 95% CI 36.2–60.6). For MDAS CR, the mean survival for ABI <0.25 was 26.5 months (SE 5.6, 05% CI 15.5–37.5), for ABI 0.25–0.89 54.0 months (SE 2.9, 95% CI 48.3–59.7), for ABI 0.9–1.29 55.7 months (SE 6.1, 95% CI 43.8–67.6), and for ABI ≥1.30 54.4 months (SE 5.0, 95% CI 44.6–64.1).

## Discussion

Based on the present observations, symptomatic LEAD appears to be multisegmental with severe infrapopliteal involvement. Together with ABI, TBI and TP should be routinely assessed from all symptomatic LEAD patients since they strongly associate with cardiovascular mortality regardless of the predominant disease location or clinical presentation.

### Multisegmental nature of LEAD

LEAD and especially CLTI, is often a multisegmental disease. Ozkan et al. angiographically assessed the overall distribution and extent of the disease; 63.9% had multisegmental disease and the most commonly affected segment was CR [14]. In angiographically evaluated CLTI patients, Gray et al. reported similar results [16]. Consistent with these studies, our results among a cohort with symptomatic LEAD patients, of which 54.5% had CLTI (Rutherford 4–6), show that symptomatic LEAD was most commonly multisegmental. The severe CR involvement was abundant even in MDAS AOI (CIx III-IV 23.3%) or MDAS FP (CIx III-IV 35.1%).

### Peripheral pressures in LEAD

Despite the multisegmental nature, the predominant disease location differs between LEAD patients. In patients with DM, distal arteries are most commonly affected whereas smoking associates with proximal LEAD [13–15]. Because of these distinctive differences between the arterial segments, it could be assumed that the outcome predictors would also vary between the territories. Although multiple studies have investigated peripheral pressures and mortality [6, 8, 9, 11, 22–25], they are not specific for arterial segments and clinical manifestation of LEAD. Previously, a decline in ABI has been interpreted as large-vessel disease progression when a reduction in TBI has been associated with progression in small vessel disease [12]. To our knowledge, this is the first study to investigate and compare ABI, TP, and TBI in different MDAS altogether and further, evaluate their impact on cardiovascular mortality.

The impact of ABI ≤0.90 on cardiovascular mortality has been widely established and an inverse correlation has been confirmed between decreased ABI and cardiovascular mortality [1, 5, 6]. In accordance with these previous studies, our results agree on these observations. The association between low ABI and cardiovascular mortality was most evident in both MDAS FP and MDAS CR. Nevertheless, ABI has some limitations; in patients with DM and CKD that commonly have CR segment involvement, the calcification of the medial layer causes incompressible arteries and incorrectly high ABI values [1].

Elevated ABI has been associated with mortality as well [5, 6, 11, 23] Singh et al. reported significantly higher overall mortality in patients with ABI >1.40 compared to ≤1.40 [23]. Hyun et al. observed an U-shaped relationship between ABI and cardiovascular mortality in diabetics [6]. In the present study, ABI >1.30 associated with cardiovascular death in MDAS FP and MDAS CR, although the finding in MDAS CR was not statistically significant in our cohort. The reason may be that although incompressible arteries elevate ABI, some patients may still have ABI within the normal range (MDAS CR, ABI 0.9–1.29, 12.0%). Supporting this, ABI levels 1.3–1.4–1.5 have been reported to have good specificity in LEAD diagnosis (86%, 94%, 96%, respectively) although the sensitivities remain modest (44%, 38%, 36%, respectively) [26].

As vessel stiffness rarely affects pedal arteries, TBI and TP may offer better risk estimation if ankle arteries are severely affected. The usefulness of TBI on cardiovascular mortality has been reported in diabetics, who often have the most extensive atherosclerosis in the CR arteries [7, 9, 27]. Irrespective of DM, a similar association between TBI and cardiovascular mortality has been reported in some studies [6, 24]. Similarly to TBI, poor TP has been associated with increased mortality as well [11, 28]. Some studies have compared TP and TBI to ABI in patients that commonly have infrapopliteal LEAD; in a dialysis patient study, of which 76% had concomitant DM, the risk of all-cause death was greater in TBI <0.7 than ABI <0.9 [8]. In a CLTI cohort, TP <10 mmHg associated with poorer survival compared to TP 31–50 mmHg whereas no statistically significant difference was detected between ABI <0.30 versus 0.31–0.5 [28].

According to our findings, extensive CR atherosclerosis was present in a considerable proportion of all MDAS groups. Inevitably, the majority of patients with extensive CR involvement (CIx III-IV, 79.4%) were in MDAS CR compared to other MDAS groups. Interestingly, severe concomitant CR involvement was not a rarity in MDAS FP (CIx III-IV 35.1) and MDAS AOI groups (CIx III-IV, 23.3%) and similarly to MDAS CR, an association between TBI, TP and cardiovascular mortality was observed. As digital arteries are often spared from arterial wall stiffening, TP and TBI seem to offer the most sensitive risk estimation in multisegmental LEAD. Based on these observations, the extent of CR atherosclerosis seems to correlate with the TP and TBI in a linear fashion.

The assessment of peripheral pressures is feasible and straightforward to estimate survival and they can be applied to everyday practice compared to invasive methods. Our present observations support the use of all three measurements, ABI, TP and TBI as they are all provide valuable risk estimation. In symptomatic LEAD, diseased CR region and medial calcification are commonly present, which may explain why TP and TBI have strong association to mortality. When acknowledging the multisegmental nature, each of these non-invasive pressure measurements should be considered when evaluating the risk for cardiovascular mortality. Especially in patients with near normal ABIs, TBI and TP may be particularly useful tools for risk estimation.

### Limitations

The cohort consisted of LEAD patients (Rutherford 2–6) with intension-to-treat (invasive imaging) and therefore, the generalizability of our results to the whole population remains uncertain. Demographic information was obtained from patients’ medical records, thus there is a heavy reliance on accurate recording of the comorbidities. Strengths of this study are the long follow-up periods and an adequately sized study cohort. ABI, TP, and TBI were registered in a vascular laboratory setting by trained nurses. Unfortunately, in MDAS AOI, unbalanced ABI categories limited the risk evaluation.

## Conclusions

As symptomatic LEAD appears to be multisegmental with severe infrapopliteal involvement, our present observations support the use of all three peripheral pressure measurements, ABI, TBI and TP in risk estimation. Despite the predominant disease location or clinical phenotype, TP and TBI have a strong association to cardiovascular mortality.
